# Ellagic Acid Inhibits Bladder Cancer Invasiveness and In Vivo Tumor Growth

**DOI:** 10.3390/nu8110744

**Published:** 2016-11-22

**Authors:** Claudia Ceci, Lucio Tentori, Maria Grazia Atzori, Pedro M. Lacal, Elena Bonanno, Manuel Scimeca, Rosella Cicconi, Maurizio Mattei, Maria Gabriella de Martino, Giuseppe Vespasiani, Roberto Miano, Grazia Graziani

**Affiliations:** 1 Department of Systems Medicine, University of Rome Tor Vergata, Rome 00173, Italy; claudiaceci@hotmail.it (C.C.); tentori@uniroma2.it (L.T.); mariagraziaatzori.2@gmail.com (M.G.A.); 2 Laboratory of Molecular Oncology, “Istituto Dermopatico dell’Immacolata”—IRCCS, Rome 00167, Italy; p.lacal@idi.it; 3 Department of Experimental Medicine and Surgery, University of Rome Tor Vergata, Rome 00173, Italy; elena.bonanno@uniroma2.it (E.B.); manuel.scimeca@uniroma2.it (M.S.); 4 “Centro di Servizi Interdipartimentale, Stazione per la Tecnologia Animale”, Department of Biology, University of Rome Tor Vergata, Rome 00173, Italy; rosella.cicconi@uniroma2.it (R.C.); mattei@uniroma2.it (M.M.); 5 Laboratory of Signal Transduction, Department of Biomedicine and Prevention, University of Rome Tor Vergata, Rome 00173, Italy; maria.gabriella.de.martino@uniroma2.it; 6 Urology Unit, Department of Experimental Medicine and Surgery, University of Rome Tor Vergata, Rome 00173, Italy; vespasiani@med.uniroma2.it (G.V.); mianor@virgilio.it (R.M.)

**Keywords:** ellagic acid, polyphenolic compounds, bladder cancer, urothelial cancer, VEGF-A

## Abstract

Ellagic acid (EA) is a polyphenolic compound that can be found as a naturally occurring hydrolysis product of ellagitannins in pomegranates, berries, grapes, green tea and nuts. Previous studies have reported the antitumor properties of EA mainly using in vitro models. No data are available about EA influence on bladder cancer cell invasion of the extracellular matrix triggered by vascular endothelial growth factor-A (VEGF-A), an angiogenic factor associated with disease progression and recurrence, and tumor growth in vivo. In this study, we have investigated EA activity against four different human bladder cancer cell lines (i.e., T24, UM-UC-3, 5637 and HT-1376) by in vitro proliferation tests (measuring metabolic and foci forming activity), invasion and chemotactic assays in response to VEGF-A and in vivo preclinical models in nude mice. Results indicate that EA exerts anti-proliferative effects as a single agent and enhances the antitumor activity of mitomycin C, which is commonly used for the treatment of bladder cancer. EA also inhibits tumor invasion and chemotaxis, specifically induced by VEGF-A, and reduces VEGFR-2 expression. Moreover, EA down-regulates the expression of programmed cell death ligand 1 (PD-L1), an immune checkpoint involved in immune escape. EA in vitro activity was confirmed by the results of in vivo studies showing a significant reduction of the growth rate, infiltrative behavior and tumor-associated angiogenesis of human bladder cancer xenografts. In conclusion, these results suggest that EA may have a potential role as an adjunct therapy for bladder cancer.

## 1. Introduction

Ellagic acid (2,3,7,8-tetrahydroxy-chromeno [5,4,3-cde] hromene-5,10-dione according to the International Union of Pure and Applied Chemistry) (EA) belongs to the family of polyphenolic compounds and is a naturally occurring hydrolysis product of ellagitannins, found in pomegranates, strawberries, raspberries, blackberries, grapes, green tea and nuts. Over the last decades, promising evidence about EA antitumor activity has been accumulated, showing that it is able to prevent tumor growth and metastasis, by inhibiting tumor cell proliferation, inducing apoptosis, breaking DNA binding to carcinogens and hampering inflammation, angiogenesis, and drug-resistance processes [1,2].

Pomegranate products are among the most promising anti-tumorigenic dietary supplements. In fact, fermentation of pomegranate juice with *Lactobacillus plantarum* increases the concentration of EA and enhances both the antimicrobial activity and anti-proliferative effects of the juice as compared to fresh unfermented pomegranate juice [3]. EA is further metabolized by intestinal flora to urolithins, a family of metabolites with different phenolic hydroxylation patterns [4] that have been recently shown to exert anticancer activity in different tumor models [5,6,7].

EA antitumor activity was initially suggested after the observation that aromatase, a key enzyme in breast cancer development which converts androgens to estrogens, is inhibited by polyphenols derived from fresh pomegranate juice [8]. Subsequently, it was demonstrated that pomegranate fruit extracts enhance the action of the anti-estrogen tamoxifen in breast cancer cells [9]. Polyphenols also inhibit the expression of genes codifying for key androgen-synthesizing enzymes and androgen receptors [10] and EA was found to affect the growth, motility and invasiveness of androgen-independent prostate cancer [11].

Antitumor activity by pomegranate juice was also reported in various in vivo murine models [12] and, in particular, EA was shown to exert in vivo therapeutic effects against colon, prostate, breast and pancreatic cancer [13,14,15,16,17].

Less characterized are, instead, the antitumor effects of EA against bladder cancer that represents the most common malignant tumor of the urinary system. Bladder tumors include non-muscle-invasive bladder cancer (NMIBC) and muscle-invasive bladder cancer (MIBC). At diagnosis, most patients (~75%) present with NMIBC and, even though the 5-year survival is >90%, the recurrence rate is high. Transurethral resection of the bladder tumor followed by intravesical instillations of mitomycin C or of Bacillus Calmette–Guérin (BCG) is the treatment of choice for NMIBC according to the risk group [18]. By contrast, MIBC has a poor outcome and requires radical cystectomy with extended lymphadenectomy, often preceded by cisplatin-based neoadjuvant chemotherapy. High-risk patients will benefit from cisplatin-containing adjuvant chemotherapy [19]. Moreover, radiotherapy is used when cystectomy is not a feasible option. Treatment of the metastatic disease relies on a combination chemotherapy protocols, including gemcitabine and cisplatin or methotrexate, vinblastine, adriamycin and cisplatin.

The high incidence and recurrence rate of NMIBC and the poor survival of MIBC with metastatic disease make bladder cancer a serious clinical need [19]. Currently, clinical trials are evaluating targeted therapies including, among others, anti-angiogenic agents, and immunotherapy with immune checkpoints inhibitors [20]. Indeed, the angiogenic factor vascular endothelial growth factor-A (VEGF-A) is highly expressed both in tumor and urine samples of bladder cancer patients and correlates with poor prognosis, being associated with progression and tumor recurrence [21]. Moreover, the U.S. Food and Drug Administration (FDA) has recently approved atezolizumab [22], a humanized monoclonal antibody against the immune checkpoint programmed cell death ligand 1 (PD-L1), for platinum-treated advanced urothelial cancer. PD-L1 is present on the surface of tumor cells and in antigen presenting cells; its binding to PD-1, expressed by activated T cells and other immune cells, generates an immunosuppressive effect, allowing tumor cells to evade immune control.

Some in vitro studies have investigated human bladder cancer cell lines sensitivity to the antiproliferative and cytotoxic effects of EA [23,24,25]. Nevertheless, no data are available about EA influence on bladder cancer cell invasiveness triggered by VEGF-A and tumor growth in vivo. On this basis, we have investigated EA anti-tumor effects on bladder cancer, using four different human cancer cell lines (T24, UM-UC-3, 5637, HT-1376). The results indicated that, besides exerting in vitro antiproliferative and apoptotic effects, EA possesses additive or synergistic growth inhibitory activity in combination with mitomycin C. Treatment with EA also inhibits tumor cell invasion of the extracellular matrix components in response to VEGF-A, likely through down-regulation of VEGF receptor type 2 (VEGFR-2) levels, and modulates the expression of the immune checkpoint protein PD-L1. Finally, results of the EA antitumor effect obtained in vitro were validated for the first time in vivo in a murine model of a human bladder cancer.

## 2. Materials and Methods

### 2.1. Cell Lines, Culture Conditions and Drugs

The human bladder carcinoma cell lines—T24, UM-UC-3, 5637 and HT-1376—were purchased from American Type Culture Collection (ATCC, Manassas, VA, USA). Cells were maintained in RPMI-1640 (Sigma-Aldrich, St. Louis, MO, USA) supplemented with 10% fetal bovine serum (FBS, Sigma-Aldrich), 2 mM L-glutamine, 100 units/mL penicillin, and 100 μg/mL streptomycin sulfate, at 37 °C in a 5% CO_2_ humidified atmosphere.

The immortalized human endothelial cell line HUV-ST was cultured in Endothelial Growth Factor Medium (EGM-2; Lonza, Verviers, Belgium) supplemented with 0.4 mg/mL geneticin and 5 µg/mL puromycin, as described [26]. The human M14 melanoma-derived clones M14-N and M14-NV, which express comparable levels of the VEGF-A co-receptor neuropilin-1 (NRP-1) but lack or express VEGFR-2, respectively, were cultured in RPMI-1640 supplemented with 0.8 mg/mL geneticin [27].

For in vitro studies, the stock solution of EA (12 mM; Biostilogit, Florence, Italy) was prepared by dissolving the drug in dimethyl sulfoxide (DMSO). The final concentration of DMSO was always ≤0.5% (*v*/*v*) and did not contribute to toxicity. Mitomycin C (ProStrakan, Galashiels, UK) stock solution (3 mM) was obtained by dissolving the drug in water.

Human umbilical vein endothelial cells (HUVEC), used as a positive control for VEGFR-2, were isolated from freshly delivered umbilical cords as previously described [28] and cultured in EGM-2, whereas the lymphoblastoid Raji (ATCC) cell line, used a as positive control for PD-L1 was maintained in RPMI-1640 culture medium.

### 2.2. Cell Proliferation Assays

Short-term and long-term effects of EA treatment on cell proliferation of bladder cancer cell lines were evaluated by using the colorimetric MTS [3-(4,5-dimethylthiazol-2-yl)-5-(3-carboxymethoxyphenyl) 2-(4-sulphophenyl)-2H-tetrazolium, inner salt] assay (CellTiter 96^®^ AQueous One Solution Cell Proliferation Assay, Promega, Madison, WI, USA), and foci formation assay, respectively. For MTS assay, cells (2000–4000 cells/well) were dispensed into flat-bottom 96-well plates, exposed to vehicle or graded concentrations of EA (5–60 μM) and grown at 37 °C in a 5% CO_2_ humidified atmosphere. Six replica wells were used for each experimental condition. After 3 (for T24, UM-UC-3 and 5637 cells) or 5 days (for HT-1376 cells, which are characterized by a lower proliferation rate), 20 μL of MTS solution was added to each well and cells were incubated at 37 °C for 2 h. The quantity of colored formazan product, deriving from the reduction of the tetrazolium compound MTS by metabolically active cells, was measured by absorbance at 490 nm (reference wavelength 620 nm) on a Multiskan™ FC Microplate Photometer (Thermo Fisher Scientific, Waltham, MA, USA).

For the foci assay, cells were plated in triplicate in 6-well plates (200 cells/well for T24, UM-UC-3 and 5637; 300 cells/well for HT-1376) and exposed to vehicle or graded concentrations of EA (1.25–40 μM). After 7–10 days of culture, colonies were fixed, stained with 0.5% crystal violet in 50% ethanol and counted, as previously described [29]. Only colonies comprising >50 cells were scored as survival colonies. Chemosensitivity was evaluated in terms of IC_50_, i.e., the concentration of the drug capable of inhibiting cell growth by 50%.

### 2.3. Apoptosis Analysis by Flow Cytometry

For cell cycle and apoptosis analysis of bladder cancer cells in response to EA, T24 cells were plated in 25 cm^2^ flasks (3 × 10^5^ cells) and exposed to EA (at the IC_50_) for 72 h. Cells were then harvested, washed twice in PBS and fixed in 70% ethanol at −20 °C for 18 h. After centrifugation, cells were resuspended in 1 mL of hypotonic solution containing 50 µg/mL propidium iodide (PI; Sigma-Aldrich), 0.1% sodium citrate (*w*/*v*), 0.1% Triton-X (*v*/*v*), and 10 µg/mL RNase (Roche, Mannheim, Germany), and incubated on ice in the dark for 30 min before fluorescence activated cell sorting (FACS) analysis. Data collection was gated utilizing forward light scatter and side light scatter to exclude cell debris and aggregates. The PI fluorescence was measured on a linear scale using a FACSscan flow cytometer (Becton Dickinson, Franklin Lakes, NJ, USA). Cell cycle analysis was performed on cell population excluding sub-G1 population and using the Mod-Fit software version 3.0 (Becton Dickinson).

### 2.4. Invasion and Migration Assays

An in vitro invasion assay was performed using the Boyden chamber and three-dimensional tumor spheroid invasion assays. Boyden chambers (Nuclepore, Whatman Incorporated, Clifton, NJ, USA) were equipped with 8-µm pore diameter polycarbonate filters (Sigma-Aldrich), coated with 20 µg of Cultrex^®^ basement membrane extract (Trevigen^®^, Gaithersburg, MD, USA) [28]. Tumor cells were suspended in RPMI-1640 medium containing 1 µg/mL heparin/0.1% fatty acid-free bovine serum albumin (BSA; Sigma-Aldrich) (hereafter referred to as invasion medium), with or without EA (at the IC_25_ for each cell line) and pre-incubated for 1 h in a rotating wheel. Cells (2 × 10^5^ in 220 µL) were then loaded into the upper compartment of the Boyden chambers. Invasion medium (200 μL), containing or not human VEGF-A (50 ng/mL; Peprotech, Rocky Hill, NJ, USA) or, in selected experiments, epidermal growth factor (EGF) (50 ng/mL; Peprotech), was added to the lower compartment of the chambers. Chambers were incubated at 37 °C in a CO_2_ incubator for 2 h to 18 h, depending on the invasive properties of the tested cell line. Filters were removed from the chambers and cells were fixed in ethanol for 5 min and stained in 0.5% crystal violet for 15 min. Non-invading cells, adherent to the upper surface of the filters, were gently removed by wiping with a cotton swab, whereas invading cells, attached to the lower surface of the filters, were counted under the microscope. Six high-magnification microscopic fields (×200 magnification), randomly selected on triplicate filters, were scored for each experimental condition. 

For spheroid invasion assay, tumor cells (25,000–30,000 cells/mL) were suspended in RPMI-1640 medium containing 10% FBS and supplemented with methyl cellulose (0.24% final concentration; Sigma-Aldrich), seeded in 96-well round bottom cell culture plates (100 μL/well; Corning^®^ Costar^®^ Ultra-Low attachment multi-well, Sigma-Aldrich) and centrifuged at 3000 rpm for 90 min [30]. Plates were then incubated for 24 h under standard culture conditions (5% CO_2_, at 37 °C) to allow spheroid formation. Spheroids were collected, embedded individually in 100 μL of 1 mg/mL collagen I solution (rat tail, Trevigen^®^), with or without VEGF-A (50 ng/mL) and/or EA, and plated in each well of a 96-well flat bottom plate, previously coated with 50 μL of collagen I. Three replicates were set up for each experimental group. Collagen I dilution and neutralization were performed according to the manufacturer’s instructions. After collagen solidification at 37 °C, 100 μL of invasion medium, with or without VEGF-A (50 ng/mL) and/or EA, were added and plates incubated overnight at 37 °C for up to 72–96 h. Spheroids were visualized and photographed using a Nikon Eclipse TS100 microscope in conjunction with a Nikon DS-Fi1 high resolution camera (Melville, NY, USA). Measurements were performed using Adobe Photoshop CS6 software. Invasion area was defined as the distance from the edge of the spheroid to the cells most distant from the spheroid.

Migration assay was performed in Boyden chambers containing gelatin (5 µg/mL; Sigma-Aldrich) coated filters and using the same experimental conditions described above for the Boyden chamber invasion assay [28].

### 2.5. Western Blot Analysis

For Western blot analysis, cells were washed with cold PBS, lysed with RadioImmunoPrecipitation Assay (RIPA) buffer (25 mM Tris-HCl pH 7.6, 150 mM NaCl, 1% NP-40, 1% sodium deoxycholate, 0.1% SDS) containing 1× cocktail protease inhibitor (Roche). Denatured proteins were separated by 8%–12% sodium dodecyl sulfate-polyacrylamide gel electrophoresis (SDS-PAGE) and then transferred to nitrocellulose membrane (GE Healthcare Life Science, Milan, Italy). Membranes were subsequently blocked in 5% non-fat dried milk in Tween-Tris-buffered saline (T-TBS) and incubated with the following primary antibodies: mouse monoclonal anti-Flk-1 (VEGFR-2, clone A3; 1:500 dilution; Santa Cruz Biotechnology, Santa Cruz, CA, USA), rabbit polyclonal anti-PD-L1 (1:300 dilution; Abcam, Cambridge, UK) or anti-β-actin (1:10,000; Sigma Aldrich) as a loading control. Goat anti-mouse or anti-rabbit Ig/Horseradish peroxidase secondary antibodies (Biorad, Hercules, CA, USA) and ECL Western blotting detection reagents (GE Healthcare Life Science) were used to identify the proteins of interest.

### 2.6. Evaluation of VEGF-A Secretion and Analysis of VEGFR-2 Phosphorylation

Semi-confluent tumor cell cultures were incubated in 0.1% BSA/RPMI-1640 medium without FBS for 24 h. Culture supernatants were collected, centrifuged at 600× *g* for 10 min to remove cells in suspension, concentrated at least ten-fold in Centriplus concentrators (Amicon, Beverly, MA, USA) and frozen at −20 °C till use. Cells were detached from the flasks with a solution of 1 mM EDTA in PBS and the total cell number/culture was recorded. Quantification of the amount of VEGF-A in the concentrated supernatants was performed using Maxisorp Nunc immunoplates (Nunc, Roskilde, Denmark) coated with goat anti-VEGF-A IgGs, as previously described [31]. Briefly, detection of the cytokines was performed with biotinylated goat anti-VEGF (R & D Systems, Abingdon, UK) and streptavidin-alkaline phosphatase conjugate (1:10,000) (Roche). The reaction was stopped and optical density at 405 nm was measured in a Microplate reader 3550-UV (Bio-Rad, Hercules, CA, USA).

Modulation of VEGFR-2 phosphorylation in response to VEGF-A in untreated cells or cells exposed to EA was analyzed using the PathScan^®^ Phospho-VEGFR-2 (Tyr1175) Sandwich Elisa Kit (Cell Signaling Technology, Danvers, MA, USA).

### 2.7. In Vivo Study

UM-UC-3 cells (5 × 10^6^) were injected intramuscularly (i.m.) in the hind leg of 5-weeks old athymic CD-1 male mice (nu/nu genotype, Charles River, Calco, Milan, Italy). Treatment with EA (40 mg/Kg) started three days after tumor challenge. The compound was dissolved in 100% DMSO to the concentration of 10 mg/mL, further diluted in saline to reach a final concentration of 1 mg/mL and administered intraperitoneally (i.p.) daily for a total of 15 days. Control mice were treated with an equivalent dilution of vehicle for 15 days.

Body weight (BW) was measured thrice-weekly and toxicity was evaluated on the basis of net BW reduction. The percentage of net BW variation between the first day of treatment and sacrifice day, was evaluated according to the following equation:

% net BW variation = [(net BW at observation day − net BW at first day of treatment)/net BW at first day of treatment] × 100(1)

Xenograft growth volume was monitored by measuring tumor mass three times a week in two dimensions by a caliper. Volumes were calculated according to the following formula:

Tumor volume (mm^3^) = [length (mm) × width^2^ (mm^2^)]/2 (2)

Antitumor efficacy of treatment with EA was assessed by the following end-points:

(a) Percentage of tumor volume inhibition (TVI%) in treated vs. control mice, calculated as:
  TVI% = 100 − [(TV treated/TV control) × 100](3)
(b) Tumor growth quadrupling time, calculated as the time required for tumor volume to increase 4-fold over initial volume at indicated times, using the formula:
  t_q_ = t_1_ + (t_2_ − t_1_)log(V_q_/V_1_)/log(V_2_/V_1_) (4)
  where t_q_ is the interpolated quadrupling time; t_1_ and t_2_ are the lower and upper observation times bracketing the quadrupling tumor volume; V_q_ = 4V_0_, where V_0_ is the initial tumor volume; V_1_ and V_2_ are tumor volumes at the times t_1_ and t_2_, respectively;
(c) Tumor growth delay index, calculated as the mean treated/control tumor growth quadrupling time ratio.

Animals were euthanized, for ethical reasons, when tumor volume was 2000 mm^3^.

At sacrifice, tumors were excised, fixed in 10% buffered formalin solution (*v*/*v*), paraffin embedded and cut into 5 µm-thick slices for staining. A set of slides was stained with hematoxylin eosin for morphological studies and mitosis counts. Additional slides were stained with an anti-mouse PECAM/CD31 polyclonal antibody (M-20, Santa Cruz Biotechnology) to label blood vessels (SP115, Abcam, Cambridge, UK). Reactions were revealed by a HRP—DAB Detection Kit (UCS diagnostic, Rome, Italy).

### 2.8. Animal Care and Ethics Statement

All procedures involving mice and care were conducted in accordance with the ethical standards, according to the Declaration of Helsinki, in compliance with our institutional animal care guidelines and following national and international directives (D.L. 4 March 2014, No. 26; directive 2010/63/EU of the European parliament and council; Guide for the Care and Use of Laboratory Animals, United States National Research Council, 2011). The animals were kept in Specific Pathogen-Free (SPF) conditions using top filter cages; sterilized tap water and food (4RFN, Mucedola, Settimo Milanese, Italy) were given ad libitum. Experimental protocols were approved by the Institutional Animal Care and Use Committee (project identification code 619/2015-PR, approved by “Organismo preposto al benessere degli animali” (O.P.B.A.), University of Rome Tor Vergata, 27-1-2015).

### 2.9. Statistical Analysis

Results were expressed as arithmetic mean ± standard deviation (SD). Difference significance was tested by an unpaired, two-tailed Student’s *t*-test. For multiple comparisons, a statistical analysis of the results was performed by ANOVA, followed by Bonferroni’s post-test. *p* values < 0.05 were considered significant.

To evaluate whether the combination of EA plus mitomycin C was synergic, cells were exposed to each drug alone or in combination with equitoxic concentrations of the drugs. The dose–effect curves were analyzed by the median-effect method of Chou and Talalay using the Calcusyn Software as a constant ratio combination (Biosoft, Cambridge, UK). The combination index (CI) indicates a quantitative measure of the degree of drug interaction in terms of synergistic (CI < 1), additive (CI = 1) or antagonistic effect (CI > 1) [32].

Immunohistochemical data were analyzed by the Mann-Whitney test (*p* < 0.0005).

## 3. Results

### 3.1. Anti-Proliferative and Apoptotic Effects of EA against Bladder Cancer Cells

The short and long-term effects of graded concentrations of EA (5–60 μM) on cell proliferation were tested in four different human bladder cancer (grade II–III) cell lines (i.e., T24, UM-UC-3, 5637, HT-1376) by MTS assay and foci assay, respectively. The results indicated that T24, UM-UC-3 and 5637 cell lines showed similar sensitivity to EA, whereas the HT-1376 cell line, characterized by a slower proliferation rate, was the most resistant (Table 1).

**Figure 1:**
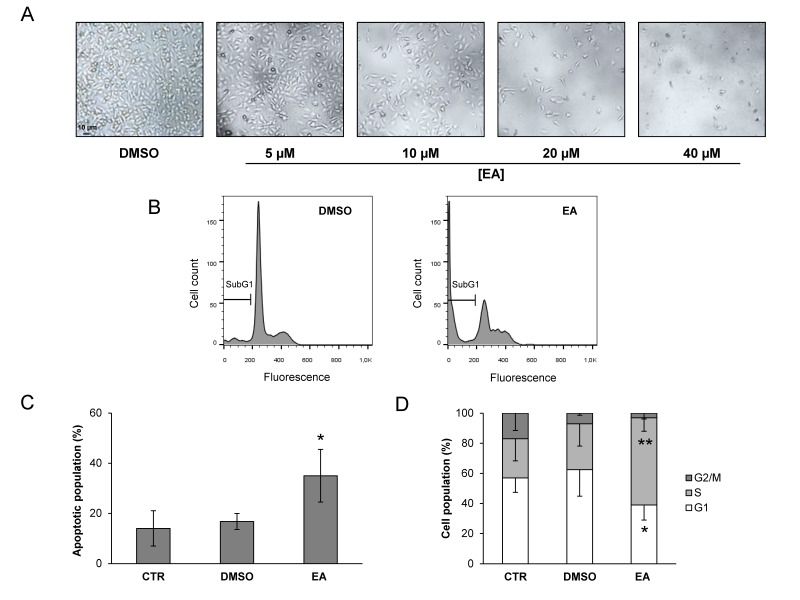
The anti-proliferative effects of EA are associated with apoptosis and S phase accumulation in bladder cancer cells. (**A**) Dose-dependent anti-proliferative effects of EA. T24 cells were exposed to a vehicle (DMSO 0.3% (*v*/*v*)) or graded concentration of EA (5–40 µM). Panels display representative images showing morphological changes in the control and EA treated cells using phase contrast microscopy (40× magnification); (**B**,**C**) Apoptosis induction in T24 cells. Cells were treated with a vehicle or 20 µM EA and analyzed by flow cytometry at 72 h. Flow cytometry plots of a representative experiment indicating subG1 apoptotic cells (**B**); Histogram represents the mean percentage values (±SD) of apoptotic cells from three independent experiments (**C**); The results of statistical analysis by the Student’s *t*-test of the differences in the percentage of apoptotic cells were as follows: EA vs. CTR (untreated cells) or DMSO, *p* < 0.05 (*); DMSO vs. CTR, not significant (NS); (**D**) Cell cycle analysis. T24 cells were treated and processed as described in panels B and C. The results are indicated as percentages of cells in the different phases of cell cycle at 72 h after treatment and are the means (−SD) from four independent experiments. Differences between the percentage of cells in S or G1 phases evaluated by the Student’s *t*-test were as follows: S phase, EA treated cells vs. CTR or DMSO, *p* < 0.01 (**); G1 phase, EA treated cells vs. CTR or DMSO, *p* < 0.05 (*).

The dose-dependent anti-proliferative activity of EA in T24 cells is shown in Figure 1A. To further characterize the inhibitory effect of EA, T24 cells were exposed to an EA concentration corresponding to the IC_50_ value (20 μM) or to vehicle and apoptosis induction and cell cycle distribution were investigated by flow cytometric analysis. Exposure to EA for 72 h resulted in induction of apoptosis (Figure 1B) and a higher percentage of cells in the S phase, compared to the vehicle treated control (Figure 1C). 

### 3.2. EA Enhances the Anti-Proliferative Effects of Mitomycin C

Mitomycin C is a chemotherapeutic agent commonly used for intravesical instillation in bladder cancer. To test whether treatment with EA might affect the response of bladder cancer cells to mitomycin C, combination studies were performed using the MTS assay. Tumor cells were initially exposed to graded concentrations of mitomycin C in order to define the IC_50_ values for each cell line. Thereafter, cells were treated with increasing equitoxic concentrations of the two compounds and combination indexes (CI) were evaluated according to the Chou–Talalay method by the CalcuSyn program. As indicated in Table 2, UM-UC-3 and 5637 cells were less sensitive to mitomycin C as compared to T24 and HT-1376 cells. Interestingly, the drug combination resulted in synergistic effects in three out of four cell lines, with CI comprised between 0.7 and 0.9. The synergistic effect was more pronounced in the HT-1376 bladder cancer cell line, which is the most resistant to EA, whereas only a weak additive effect was detected in T24 cells. Interestingly, an analysis of the dose reduction index (DRI) indicated that addition of EA allowed up to a 2.6-fold reduction of mitomycin C IC_50_. These data represent the first evidence of the feasibility of using EA treatment to potentiate the antitumor activity of mitomycin C.

### 3.3. Ellagic Acid Reduces VEGFR-2 Expression in Human Bladder Cancer Cells

**Figure 2:**
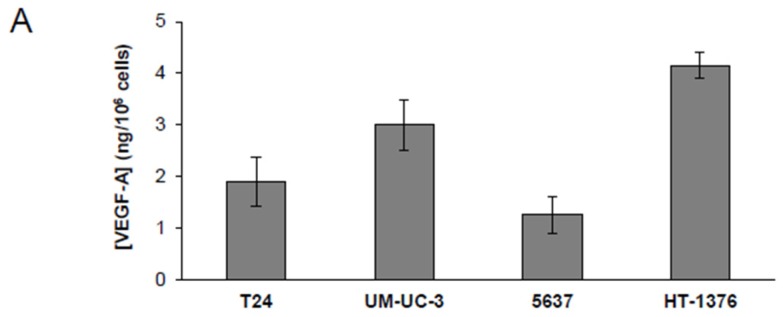

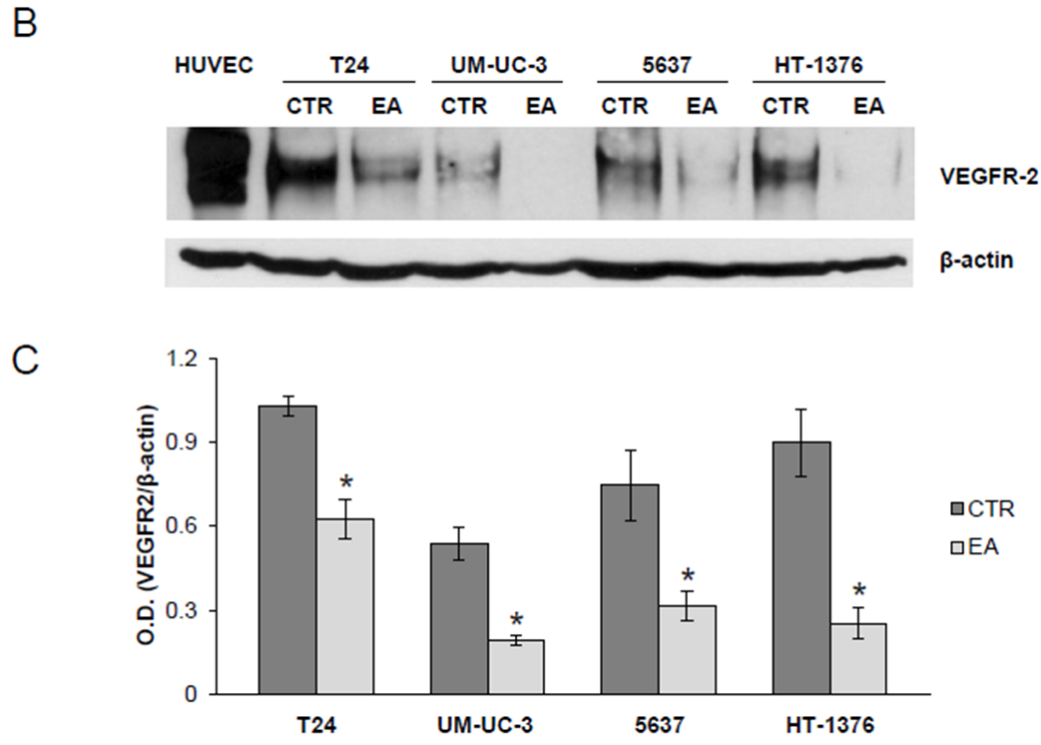
Treatment with EA reduces VEGFR-2 expression. (**A**) VEGF-A levels released in tumor cell culture supernatants. Quantification of the amount of VEGF-A in the concentrated supernatants of bladder cancer cell lines was performed using Maxisorp Nunc immunoplates coated with goat anti-VEGF-A IgGs. Results are the mean (± SD) of three independent determinations; (**B**) Immunoblot analysis of VEGFR-2. Western blot analysis of the levels of VEGFR-2 expressed in control (CTR) and in bladder cancer cell lines, treated with a vehicle (CTR) or exposed to EA for 24 h, at concentrations in the range of IC_50_ values for each cell line (i.e., 20 µM, T24; 40 µM, UM-UC-3; 27 µM 5637; 60 µM HT-1376). HUVEC were loaded as a positive control and β-actin as a loading control; (**C**) Densitometric analysis. The relative levels of VEGFR-2 were calculated by densitometric analysis and normalized using β-actin expression in each sample. The histogram represents the ratios between the optical densities (O.D.) of VEGFR-2 in CTR or EA treated groups and β-actin. Results are the mean (±SD) of three independent experiments. Student’s *t*-test analysis: EA vs. CTR, *p* < 0.05 (*).

Previous studies showed that EA exerts anti-angiogenic effects in breast cancer inhibiting VEGFR-2 signaling; these findings were supported by the results of an in silico analysis suggesting a possible interaction between VEGFR-2 and the polyphenol [16]. Moreover, EA has been reported to inhibit VEGFR-2 expression in pancreatic cancer cells [17], whereas it did not induce any change in the receptor levels in breast cancer cells [16]. Here, we have investigated whether the bladder cancer cell lines tested in our study secrete VEGF-A, express VEGFR-2 and undergo modulation of the receptor when treated with EA. The results indicated that all cell lines secreted detectable amounts of VEGF-A in culture supernatants (Figure 2A) and expressed VEGFR-2, albeit to a different extent (Figure 2B,C). Interestingly, treatment with EA IC_50_ for 24 h induced a significant reduction of receptor expression, with percentages of inhibition ranging between 39% and 72% depending on the cell line tested (Figure 2B,C). However, EA did not affect VEGF-A induced phosphorylation of VEGFR-2.

### 3.4. EA Inhibits Extracellular Matrix Invasion and Migration of Human Bladder Cancer Cells in Response to VEGF-A

**Figure 3:**
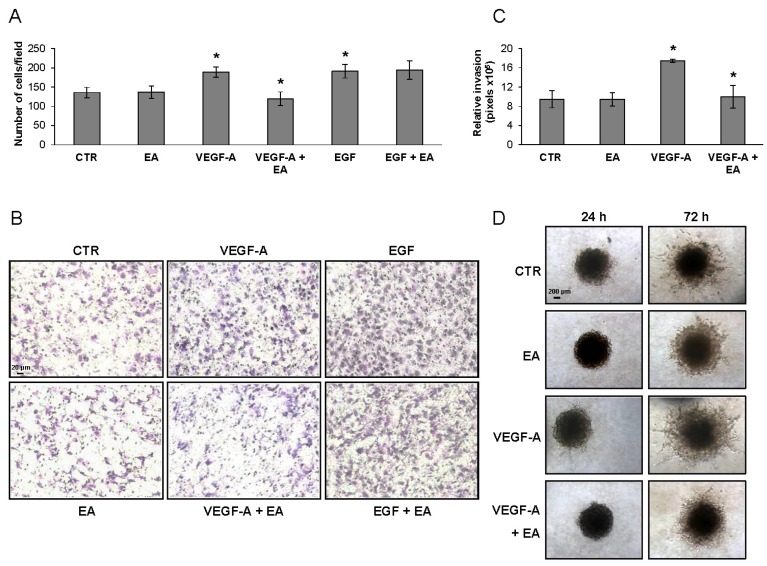
Treatment with EA inhibits T24 bladder cancer cell invasion in response to VEGF-A, but not to EGF. Invasion of T24 cells (2 × 10^5^ cells/chamber, 2 h incubation), non-stimulated (CTR) or exposed to EA IC_25_ (10 µM) in response to VEGF-A (50 ng/mL) or to EGF (50 ng/mL), was tested in Boyden chambers containing matrigel coated filters. Invading cells were counted in six random microscopic fields for each experimental condition. The histogram represents the arithmetic mean values of migrated cells/microscopic field ± SD of three independent determinations. Results of the statistical analysis performed by one-way ANOVA, followed by Bonferroni’s post-test for multiple comparison, were as follows: VEGF-A vs. CTR or EA, *p* < 0.05; VEGF-A + EA vs. VEGF-A, *p* < 0.05; EGF vs. CTR or EA, *p* < 0.05 (*); EGF + EA vs. EGF, NS (**A**); Photographs from a representative experiment out of three are shown (×100 magnification) (**B**); For the spheroid assay, T24 cells were embedded in collagen in the absence or presence of EA (10 µM) and VEGF-A (50 ng/mL). Relative invasion was quantified as spheroid area on day 3 minus spheroid area on day 0. Results are expressed as mean ± SD of quadruplicate samples. Results of the statistical analysis using one-way ANOVA, followed by Bonferroni’s post-test, were as follows: VEGF-A vs. CTR or EA, *p* < 0.05; VEGF-A + EA vs. VEGF-A, *p* < 0.05 (*) (**C**). Representative pictures of spheroids taken at 24 and 72 h after embedding cells in collagen gels (×40 magnification) (**D**).

The EA influence on extracellular matrix invasion in response to VEGF-A or to EGF (a VEGFR-2 unrelated stimulus) by bladder cancer cells was tested in Boyden chambers endowed with filters coated with matrigel. Invasion of T24 cells exposed to VEGF-A was strongly down-modulated by EA at mildly toxic concentrations (EA IC_25_), whereas invasion triggered by EGF was not affected (Figure 3A,B). The ability of EA to hinder invasiveness of VEGFR-2 expressing bladder cancer cells in response to VEGF-A was also confirmed using an in vitro three-dimensional spheroid-based assay in the collagen I matrix (Figure 3C,D).

**Figure 4:**
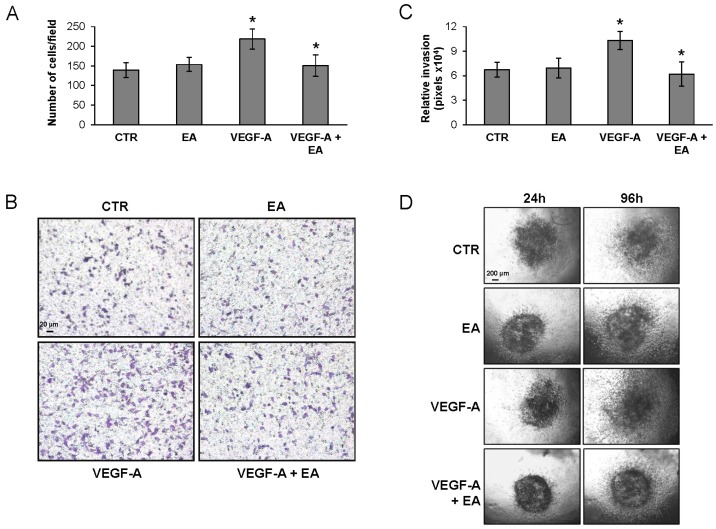
Inhibitory effect of EA on UM-UC-3 cell invasion in response to VEGF-A. Matrigel invasion assay. Invasion of UM-UC-3 cells (2 × 10^5^ cells/chamber, 4 h incubation), non-stimulated (CTR) or exposed to EA IC_25_ (20 µM) in response to VEGF-A (50 ng/mL) was tested in Boyden chambers containing matrigel coated filters (**A**,**B**) or by spheroid invasion assay (**C**,**D**). For matrigel invasion test, invading cells were counted in six random microscopic fields for each experimental condition. Histogram represents the arithmetic mean values of migrated cells/microscopic field ± SD of three independent determinations. Results of the statistical analysis using one-way ANOVA, followed by Bonferroni’s post-test, were as follows: VEGF-A vs. CTR or EA, *p* < 0.05; VEGF-A + EA vs. VEGF-A, *p* < 0.05 (*) (**A**); Photographs from a representative experiment out of three are shown (×100 magnification) (**B**); For spheroid invasion assay, UM-UC-3 cells were embedded in collagen in the absence or presence of EA (20 µM) and VEGF-A (50 ng/mL). Data are expressed as mean ± SD of quadruplicate samples. Results of the statistical analysis using one-way ANOVA, followed by Bonferroni’s post-test, were as follows: VEGF-A vs. CTR or EA, *p* < 0.05; VEGF-A + EA vs. VEGF-A, *p* < 0.05 (*) (**C**); Representative pictures of spheroids taken at 24 and 96 h after embedding cells in collagen gels (×40 magnification) (**D**).

**Figure 5:**
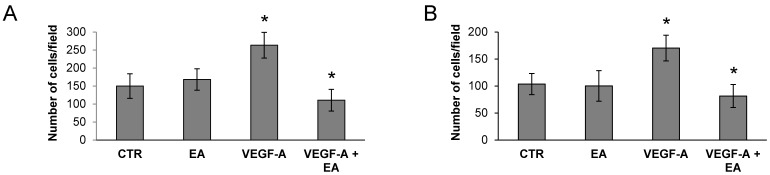

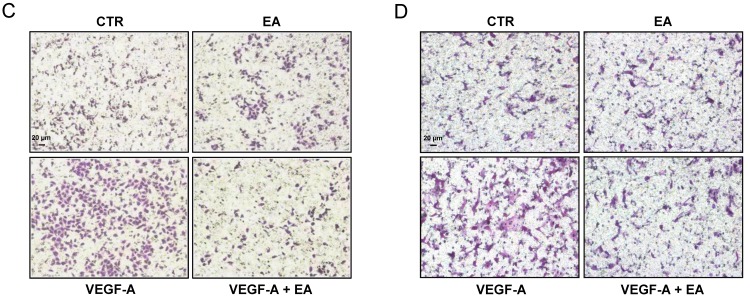
Inhibitory effect of EA on 5637 and HT-1376 cell invasion in response to VEGF-A. Invasion of 5637 (**A**,**C**) and HT-1376 (**B**,**D**) cells (2 × 10^5^ cells/chamber, 18 h incubation), non-stimulated (CTR) or exposed to EA IC_25_ of each cell line (13.5 µM for 5637 cells and 30 µM for HT-1376) in response to VEGF-A (50 ng/mL) was tested in Boyden chambers containing matrigel coated filters. Invading cells were counted in six random microscopic fields for each experimental condition. Histograms represent the arithmetic mean values of migrated cells/microscopic field ± SD of three independent determinations. Results of the statistical analysis using one-way ANOVA, followed by Bonferroni’s post-test, were as follows for both cell lines: VEGF-A vs. CTR or EA, *p* < 0.05; VEGF-A + EA vs. VEGF-A, *p* < 0.05 (*) (**A**,**B**). Photographs from a representative experiment out of three are shown (×100 magnification) (**C**,**D**).

**Figure A1:**
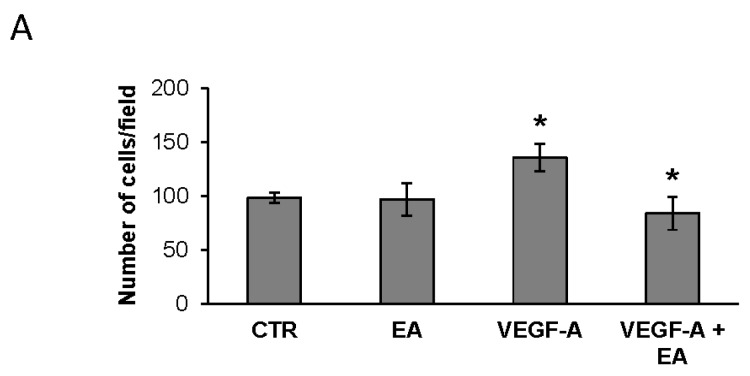

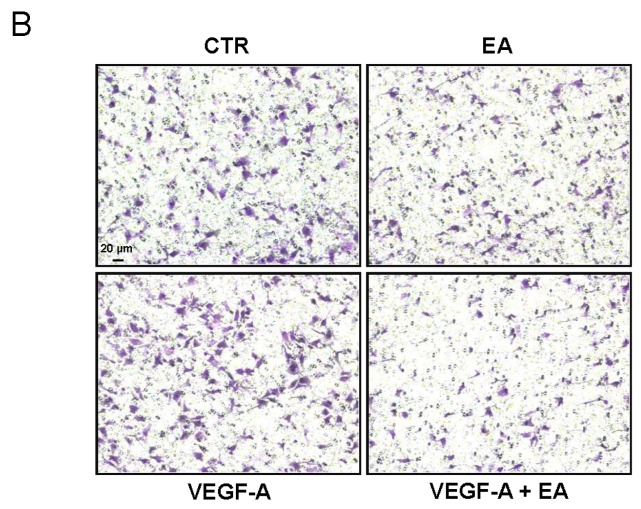
Treatment with EA inhibits matrigel invasion by endothelial cells. Invasion of HUV-ST endothelial cells (2 × 10^5^ cells/chamber, 3 h incubation), non-stimulated (CTR) or exposed to EA IC_25_ (20 µM) in response to VEGF-A (25 ng/mL) was tested in Boyden chambers containing matrigel coated filters. Invading cells were counted in six random microscopic fields for each experimental condition. Histogram represents the arithmetic mean values of migrated cells/microscopic field ± SD of three independent determinations. Results of statistical analysis using one-way ANOVA, followed by Bonferroni’s post-test, were as follows: VEGF-A vs. CTR or EA, *p* < 0.05 (*); VEGF-A + EA vs. VEGF-A, *p* < 0.05 (*) (**A**); Photographs from a representative experiment out of three are shown (×100 magnification) (**B**).

**Figure A2:**
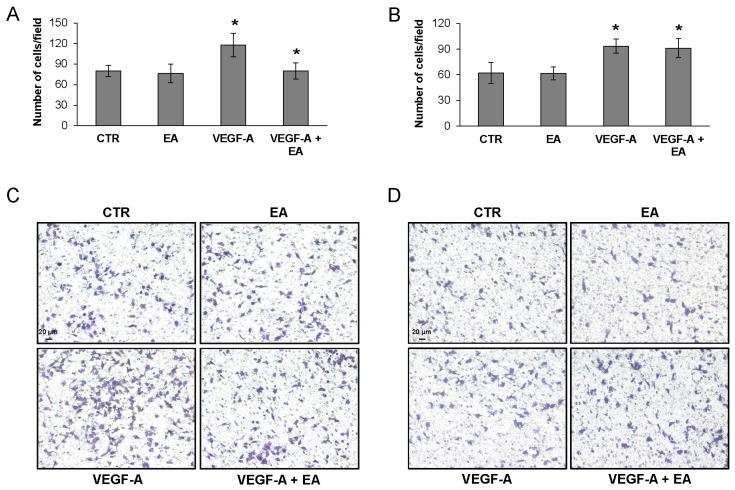
VEGFR-2 expression is required for the inhibitory effect of EA on tumor cell invasiveness. Invasion of M14-NV (VEGFR-2 positive) (**A**,**C**) and M14-N (VEGFR-2 negative) (**B**,**D**) melanoma cell clones (2 × 10^5^ cells/chamber, 2 h incubation), non-stimulated (CTR) or exposed to EA IC_25_ of each cell line (15 µM) in response to VEGF-A (50 ng/mL) was tested in Boyden chambers containing matrigel coated filters. Invading cells were counted in six random microscopic fields for each experimental condition. Histograms represent the arithmetic mean values of migrated cells/microscopic field ± SD of three independent determinations. Results of statistical analysis performed by one-way ANOVA, followed by Bonferroni’s post-test for multiple comparison, were as follows: in M14-NV cells VEGF-A vs. CTR or EA, *p* < 0.05; VEGF-A + EA vs. VEGF-A, *p* < 0.05 (*); in M14-N cells VEGF-A or VEGF-A + EA vs. CTR or EA, *p* < 0.05; VEGF-A + EA vs. VEGF-A, NS (**A**,**B**); Photographs from a representative experiment out of three are shown (×100 magnification) (**C**,**D**).

Consistently, EA markedly reduced invasiveness of UM-UC-3 (Figure 4), 5637 and HT-1376 cells (Figure 5). Similar EA concentrations also hampered extracellular matrix invasion in response to VEGF-A by human endothelial cells (Figure A1) which express VEGFR-2 [26]. The requirement of VEGFR-2 expression for EA inhibitory effects on extracellular matrix invasion stimulated by VEGF-A was confirmed in a syngeneic model of human melanoma, using VEGFR-2-negative and -transfected clones (M14-N and M14-NV, respectively) [27]. In fact, even though invasiveness of M14-N cells, which express the VEGF-A co-receptor NRP-1 but lack VEGFR-2, was stimulated by VEGF-A, EA inhibited VEGF-A induced extracellular matrix invasion only by VEGFR-2 positive M14-NV cells, producing no effects on M14-N cells (Figure A2).

**Figure 6:**
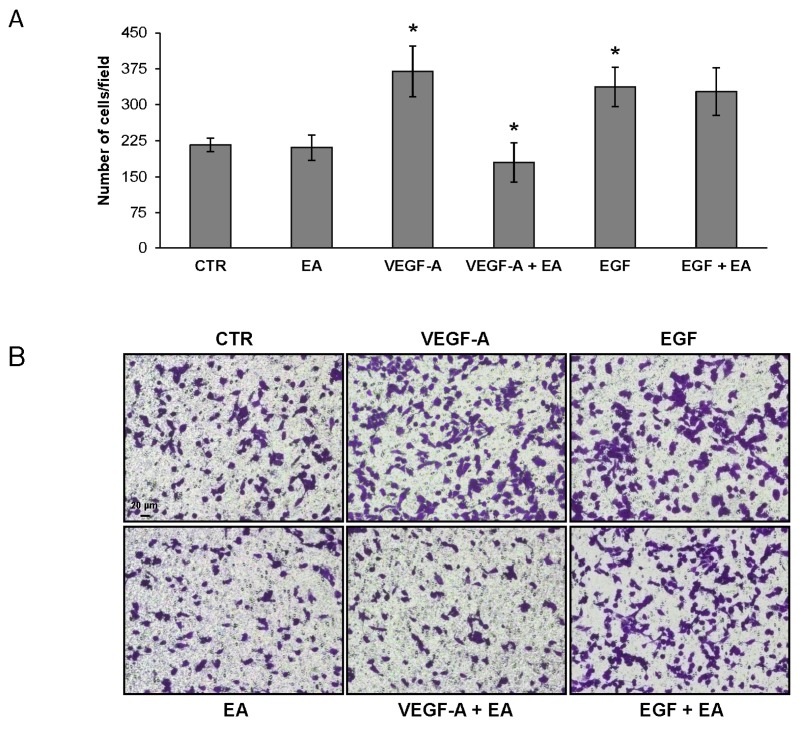
Treatment with EA inhibits migration of UM-UC-3 cells in response to VEGF-A but not to EGF. Migration of UM-UC-3 cells (2 × 10^5^ cells/chamber, 18 h incubation), non-stimulated (CTR) or exposed to EA IC_25_ (20 µM) in response to VEGF-A or EGF (50 ng/mL) was tested in Boyden chambers containing gelatin coated filters. Migrating cells were counted in six random microscopic fields for each experimental condition. The histogram represents the arithmetic mean values of migrated cells/microscopic field ± SD of three independent determinations. Results of the statistical analysis using one-way ANOVA, followed by Bonferroni’s post-test, were as follows: VEGF-A vs. CTR or EA, *p* < 0.05; VEGF-A + EA vs. VEGF-A, *p* < 0.05; EGF vs. CTR or EA, *p* < 0.05 (*); EA + EGF vs. EGF, NS (**A**); Photographs from a representative experiment out of three are shown (×100 magnification) (**B**).

We also investigated the chemotactic response of bladder cancer cells to VEGF-A in Boyden chambers endowed with gelatin coated filters. A down-modulating effect of EA on the migratory response of UM-UC-3 cells to VEGF-A was observed, whereas such effect was not observed when tumor cells were exposed to a VEGFR-2 unrelated stimulus like EGF (Figure 6).

### 3.5. Treatment of Bladder Cancer Cells Decrease the Expression of PD-L1

**Figure 7:**
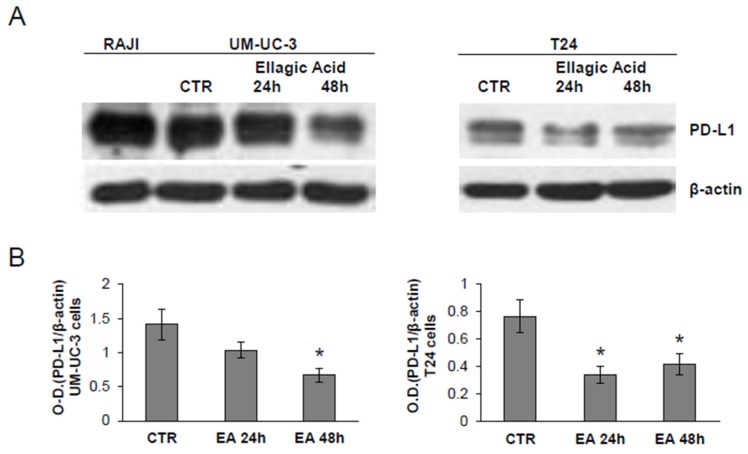
Treatment with EA reduces PD-L1 expression. (**A**) Western blot analysis of PD-L1 expression in UM-UC-3 and T24 bladder cancer cells, treated with DMSO vehicle (CTR) or exposed to EA for 24 or 48 h, at a concentration in the range of IC_50_ values for each cell line (i.e., 20 µM, T24; 40 µM, UM-UC-3). Raji leukemia cells were loaded as a positive control and β-actin as loading control; (**B**) Densitometric analysis. The relative levels of PD-L1 in UM-UC-3 and T24 cells, respectively, were calculated by densitometric analysis and normalized by β-actin expression in each sample. Histograms represent the ratios between the O.D. of PD-L1 in CTR or EA treated groups and β-actin. Data are the mean (±SD) of three independent experiments. Results of the statistical analysis using one-way ANOVA, followed by Bonferroni’s post-test, were as follows: in T24 cells, EA 24 h vs. CTR, *p* < 0.05; in UM-UC-3 and T24 cells, EA 48 h vs. CTR, *p* < 0.05 (*).

Based on the recent approval by FDA of atezolizumab [22], a humanized monoclonal antibody against the immune checkpoint PD-L1, for platinum-treated advanced urothelial cancer, we have investigated the influence of EA on the expression of PD-L1. Immunoblot analysis in UM-UC-3 and T24 cells showed that EA down-modulated PD-L1 expression in both cell lines (Figure 7). These data suggest that EA might contribute to reducing tumor immune escape mechanisms that favor disease progression, potentially enhancing the efficacy of anti-PD-L1 agents.

### 3.6. EA Inhibits In Vivo Bladder Cancer Growth

**Figure 8:**
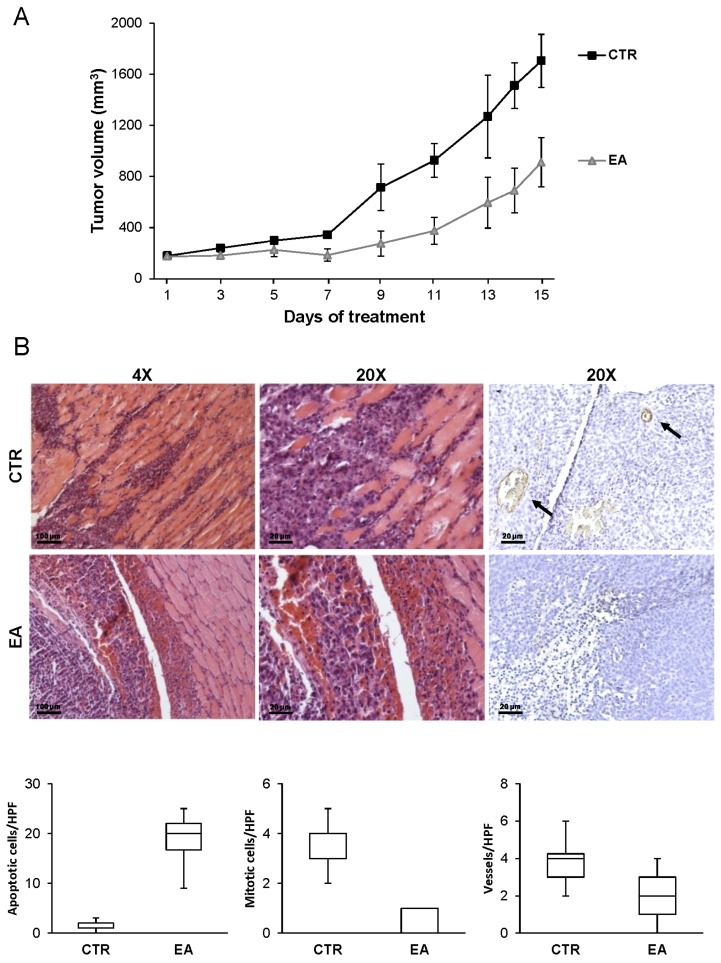
Treatment with EA inhibits the growth of human bladder xenografts in vivo. UM-UC-3 cells (5 × 10^6^) were injected in CD1 nude mice. After tumor challenge, mice were randomized and treated with a vehicle (CTR) or EA (40 mg/kg/die) (seven animals/group). (**A**) EA inhibits tumor growth in vivo. Tumor growth was evaluated every other day, as described in Materials and Methods. Results are the arithmetic mean tumor volumes ± SD. A statistical analysis was performed by Student’s *t*-test analysis. The differences in tumor sizes between the control and EA treated mice were statistically significant from day 7 onward (*p* < 0.05); (**B**) EA induces apoptosis and inhibits angiogenesis in bladder cancer in vivo. Morphology of tumor xenografts was evaluated after hematoxylin and eosin staining of histological sections obtained from control or EA treated mice. A massive cancer infiltration in the surrounding tissue is observed in CTR tumor sections, which is markedly decreased in EA treated samples (4× and 20×). Mitoses and apoptosis were analyzed by counting the number of mitotic figures or apoptotic cancer cells in 20 high power fields (HPF) at 40× magnification. Vessel formation was analyzed by immunohistochemical staining of tumor sections with an anti-mouse PECAM/CD31 polyclonal antibody and by counting the number of CD31 positive vessels in 20 HPF at 40× magnifications. Images display blood vessels in a sample of EA treated xenograft sections (arrows; 20×) and rare/absent blood vessels in a section from an EA treated mouse (20×). Box and whisker plots represent the data obtained from three mice for each experimental group (three serial sections/animal). The top and bottom of each box represent the 75th and 25th percentile, respectively, and whiskers the 10th and 90th percentiles. Results of the statistical analysis using the Mann-Whitney test were as follows: EA vs. CTR, *p* < 0.0001 (***).

EA effect on in vivo tumor growth was evaluated by injecting i.m. UM-UC-3 cells in athymic nude mice. Animals were treated i.p. with 40 mg/kg of EA or with vehicle daily, for a total of 15 days. A strong reduction in tumor volume was observed in animals treated with EA as compared to control mice, with a maximum tumor growth inhibition of 61% (Figure 8A). EA administration significantly increased the tumor growth quadrupling time (EA 12.2 ± 1 days vs. CTR 8.6 ± 1), with a tumor growth delay index of 1.42 ± 0.09. Importantly, daily administration of EA was well tolerated since no significant changes in body weight (<5%) were observed during mice treatment, as compared with control animals treated with vehicle.

Two weeks after treatment start, three mice for each experimental group were sacrificed for tumor histological analysis. Hematoxylin and eosin staining indicated that 40 mg/kg of EA had a marked effect on UM-UC-3 cell viability, the number of apoptotic cells being significantly higher than in control animals. Moreover, tumor sections from EA treated animals showed reduced mitotic activity compared with control sections. Immunohistochemical analysis revealed a significant decrease of blood vessel formation in tumor sections obtained from EA treated mice compared with control sections (Figure 8B).

## 4. Discussion

EA is a dietary-derived polyphenol which has been reported to possess anti-cancer properties. In this study, we demonstrate for the first time that EA, besides exerting anti-proliferative and apoptotic effects, inhibits invasion of the extracellular matrix in response to VEGF-A by human bladder cancer cells. Importantly, in vivo treatment of athymic nude mice significantly reduced tumor size, infiltration of surrounding tissues and neovessel formation within the tumor mass.

The majority of deaths from MIBC occur in patients with metastatic disease [33] and this appears to be due to an early spreading of tumor cells during the natural history of the disease [34]. Unfortunately, the 5-year survival of patients with metastases is only ~8% compared with ~90% of patients with localized disease. Angiogenic factors, and in particular VEGF-A, have been shown to play a relevant role in the progression of bladder cancer, being able to stimulate the recruitment of circulating endothelial progenitors from bone marrow, proliferation and migration of endothelial cells and formation of new capillary vessels within the tumor [35,36]. Indeed, the humanized monoclonal antibody anti-VEGF-A bevacizumab has shown promising activity in clinical trials against bladder cancer [37]. Here, we report that EA is able not only to inhibit human endothelial cell invasion of the extracellular matrix triggered by VEGF-A, but also to directly hamper bladder cancer cell invasive behavior. Stimulation of tumor cell invasiveness by the angiogenic factor and the inhibitory effect of EA required the expression of VEGFR-2. In fact, tumor cells lacking VEGFR-2 did not respond to VEGF-A and EA did not affect their background extracellular matrix invasion. The anti-angiogenic properties of EA have been previously demonstrated in several models of solid tumors (reviewed in 1). In a breast cancer preclinical model, EA was found to exert anti-angiogenic activity likely as a result of a possible interaction of the compound with the ATP-binding region of the catalytic domain of VEGFR-2 kinase [16]. However, in bladder cancer cells, EA did not inhibit VEGFR-2 auto-phosphorylation stimulated by VEGF-A, whereas it caused down-modulation of the receptor, as previously observed in a pancreatic cancer model [17]. The mechanisms responsible for EA-induced VEGFR-2 down-regulation have not been specifically addressed in this study. However, it can be speculated that EA might promote receptor degradation due to its possible interaction with VEGFR-2 [16] or decrease receptor synthesis, based on the recently reported ability of EA to modulate gene expression [38]. VEGFR-2 expression has been previously found to correlate with bladder cancer progression and poor prognosis in patients [39]. Moreover, immunostaining of cancer specimens has recently shown that VEGFR-2 expression is higher in MIBC than in NMIBC, suggesting that VEGFR-2 levels increase with tumor invasion [40]. Actually, the four tumor cell lines used in this study were models of bladder cancer at an advanced stage and all of them were positive for VEGFR-2 expression and produced VEGF-A. Moreover, VEGF-A was able to increase bladder cancer cell invasiveness. Our findings are in line with those reported for other tumor cell types, including bladder cancer [41,42,43]. Tumor cell exposure to EA specifically inhibited tumor invasion in response to VEGF-A. In fact, the polyphenol was unable to counteract the stimulatory effect induced by a VEGFR-2 unrelated stimulus, such as EGF.

Interestingly, EA down-regulated the expression of the immune checkpoint PD-L1 in tumor cells, suggesting that EA might contribute to reducing immunosuppressive mechanisms that favor disease progression. Moreover, it can be hypothesized that EA might potentiate the immunostimulating activity of atezolizumab, the anti-PD-L1 agent recently approved for the metastatic disease [44].

Daily administration of EA to animal injected with bladder cancer cells was well tolerated and induced a significant tumor growth inhibition. This effect was associated with apoptosis induction, decrease of tumor cell mitotic activity and reduced infiltration of the surrounding tissues. Cell death and reduced proliferation of bladder cancer cells observed in vivo might be the consequence of the direct cytotoxic and cytostatic effects of EA and of the reduced formation of vascular structures within the tumor mass. These data support the potential role of EA in reducing the metastatic potential of bladder cancer and in enhancing the efficacy of anti-VEGF-A therapies. In regard to the EA dose associated with in vivo anticancer properties, it appears largely higher than that administered as dietary supplement in humans. It should be noted that EA pharmacokinetics is quite unfavorable, due to low bioavailability as a result of poor absorption, metabolism by intestinal microorganism and short plasma half-life [45,46]. Nevertheless, future development of EA derivatives or formulations with improved pharmacokinetics may allow to better define the dose-effect relationship and to reduce the amount of the active principle to be administered in patients by systemic or local routes.

Although most patients with NMIBC generally have favorable outcomes, local therapy with chemotherapy mainly based on mitomycin C often requires repeated treatments [47]. Interestingly, we found that EA enhanced the antiproliferative effects of mitomycin C. These data suggest that also a less aggressive disease might benefit from local or systemic therapy with this compound, possibly reducing the frequency of administration of the chemotherapeutic agent.

## 5. Conclusions

In conclusion, EA displays marked in vitro and in vivo antitumor activity against human bladder cancer, as a result of different effects: inhibition of tumor cell proliferation; migration and invasion of the extracellular matrix in response to VEGF-A; down-modulation of PD-L1; and decreased tumor-associated angiogenesis. Therefore, nutrients rich in EA or dietary supplements based on EA can be a useful support for the prevention and/or treatment of bladder cancer.

## Figures and Tables

**Table 1:** Antiproliferative effects of EA.

Cell Line	EA IC_50_ MTS Assay (μM) ^a^	EA IC_50_ Foci Assay (μM) ^a^
T24	21 ± 1.5	7.1 ± 0.5
UM-UC-3	37.8 ± 0.7	7.9 ± 1.3
5637	26.7 ± 1.8	7.6 ± 1.1
HT-1376	58.8 ± 3.4	19.1 ± 0.5

^a^ Control cells were exposed to a vehicle and the percentage inhibition values used for the calculation of IC_50_s in EA treated samples were evaluated with respect to a DMSO treated control. In all cell lines, DMSO caused ≤5% inhibition of cell proliferation as compared to untreated cells. Values are the mean ± SD of three independent experiments.

**Table 2:** Antiproliferative effects of mitomycin C as a single agent or in combination with EA.

Cell Line	IC_50_ Mitomycin C ^a^ (nM)	Mitomycin C (M)+ EA CI at Different FA(%) ^b^			Dose Reduction Index (DRI) ^c^
		40	50	60	
		(M nM + EA µM)			
T24	203 ± 7.5	1 (92 + 10)	1.1 (138 + 15)	1.2 (185 + 18)	1.9
UM-UC-3	504.9 ± 89.5	0.9 (185 + 15)	0.9 (270 + 20)	0.9 (360 + 25)	2.3
5637	451.2 ± 52.4	0.8 (180 + 10)	0.8 (270 + 15)	0.8 (360 + 20)	2.2
HT-1376	273 ± 34.1	0.9 (90 + 7.5)	0.7 (130 + 15)	0.7 (240 + 30)	2.6

^a^ IC_50_s were evaluated by the MTS assay. Values are the mean ± SD of three independent experiments; ^b^ Combination index (CI) at the indicated fractions affected (FA) was evaluated according to the Chou–Talalay method combining equitoxic concentrations of mitomycin C and EA; ^c^ The DRI values refer to the fold decrease of mitomycin C IC_50_s obtainable when the drug was combined with EA.
